# Thyroid disease in the time of COVID-19

**DOI:** 10.1007/s12020-020-02364-8

**Published:** 2020-06-07

**Authors:** Dorota Dworakowska, Ashley B. Grossman

**Affiliations:** 1 Departament of Hypertension and Diabetes, Medical University of Gdansk, Gdansk, Poland; 2 Guys Richard Dimbleby Department of Cancer Research, Kings College London, London, UK; 3 London International Clinic, London, UK; 4 Centre for Endocrinology, Barts and the London School of Medicine, Queen Mary University of London, London, UK; 5 OCDEM, University of Oxford, Oxford, UK

**Keywords:** Thyroid, COVID-19, Thyroid autoimmune disease, AITD

## Abstract

The novel coronavirus disease COVID-19 is produced by SARS-CoV-2. WHO has declared COVID-19 as a public health emergency, with the most susceptible populations (requiring ventilation) being the elderly, pregnant women and people with associated co-morbidities including heart failure, uncontrolled diabetes, chronic obstructive pulmonary disease, asthma and cancer. However, such general guidance does not provide information regarding COVID-19 risks in patients with suffering from pre-existing thyroid problems, and furthermore, we do not know whether patients with COVID-19 (symptomatic or without symptoms), who have not previously had thyroid issues develop endocrine thyroid dysfunction after infection. The *European Society for Endocrinology* recently published a statement on COVID-19 and endocrine diseases (*Endocrine*, 2020); however, thyroid diseases were not mentioned specifically. We have therefore reviewed the current literature on thyroid diseases (excluding cancer) and COVID-19, including data from the previous coronavirus pandemic caused by the SARS-associated coronavirus (SARS-CoV), a member of the same family Coronaviridae leading to severe acute respiratory syndrome (SARS). At the moment there are no data suggesting that thyroid patients are at higher risk of COVID-19, but this requites further research and data analysis.

WHO declared coronavirus COVID-19 disease (caused by SARS-CoV-2) a public health emergency, with the most susceptible populations being the elderly, pregnant women and people with associated co-morbidities including heart failure, diabetes, asthma or cancer. In many countries, severe measures have been put in place concentrating on self-isolation and ‘lockdown’ to slow down disease spread and prevent loss of life [1–5].

However, such general guidance does not provide information regarding COVID-19 risks in patients with pre-existing thyroid problems. We also do not know whether COVID-19 patients, symptomatic or without symptoms, develop de novo thyroid dysfunction after infection.

Autoimmune thyroid disease (AITD) remains very common, and patients with both under- and overactive thyroid turn to their physicians for guidance if they belong to a high-risk group. In the UK there are on-going discussions with employers which may have a significant impact on workers being required to self-isolate (and work from home when possible), or to continue to come to the workplace, especially in the group of patients on anti-thyroid medications who were warned about the possible side-effect of agranulocytosis and advised that, should they have fever or a sore throat, they should seek immediate medical attention. Difficulties in accessing specialist care result in further anxiety. Patients are also not sure if the benefit from blood monitoring to adjust thyroid medications overwhelms the risk of COVID-19 exposure. Currently in the UK, patients with COVID-19 symptoms are advised to self-isolate: so, if such patients suffer also from Graves’ disease, they are unable to attend for ‘routine’ blood tests. A new way of care delivery via remote consultations is being increasingly used (over the telephone or video) and has been endorsed (when appropriate) by the UK General Medical Council (https://www.gmc-uk.org/ethical-guidance/ethical-hub/remote-consultations) [6].

Endocrine and diabetes societies have responded to COVID-19 by producing guidance in cortisol-deficient patients (due to primary adrenal failure or hypopituitarism), patients treated with glucocorticoids for underlying inflammatory conditions, and in patients with diabetes [7, 8]. However, patients with thyroid disorders have not been given specific advice.

The *European Society for Endocrinology* recently published a statement in the journal *Endocrine* with the following recommendations to adequately protect the patient and ask for COVID-19 testing if exposed, to avoid unnecessary routine appointments in person but to put in place online/email/phone consultation services. For patients with diabetes the recommendation was made to closely monitor glycaemic control and strictly adhere to ‘sick day rules’ in case of COVID-19 infection. For hypo-adrenal patients, close clinical monitoring and the need for increased replacement therapy (if clinically indicated) was recommended [9]. Nevertheless, a specific focus on patients with thyroid disorders is lacking.

To the best of our knowledge, there are no papers published so far specifically on thyroid/AITD and COVID-19. However, some analogies and assistance may be drawn from the rheumatology experience which also deals with autoimmune diseases.

Thyroid abnormalities may represent an isolated alteration, but they also may be the harbinger of a future autoimmune polyglandular syndrome [10]. They may also precede or follow connective tissue diseases or rheumatoid arthritis (RA). The mechanisms by which AITD may be linked to systemic autoimmune diseases have not yet been fully understood [11, 12]. However, it has been suggested that RA patients do not belong to a high-risk group for COVID-19, and since immunological dysfunction has features related to AITD, one may infer that this is likely to also be the case for AITD [13].

The question regarding the risk of the potential use of anti-thyroid medication during the COVID-19 pandemic is more difficult to answer, and a precautionary approach might be wise, while awaiting further information. Non-chemotherapy idiosyncratic drug-induced neutropenia (IDIN) is a relatively rare but potentially fatal disorder that occurs in susceptible individuals, with an incidence of 2.4–15.4 cases per million population/year (summarised by Curtis) [14]. Affected patients typically experience severe neutropenia within several weeks and up to several months after first exposure to a drug: the mortality is ∼5%. The anti-thyroid drugs most frequently associated with IDIN include thiamazole (methimazole) and carbimazole. Laboratory testing for neutrophil drug-dependent antibodies is rarely performed because of the complexity and low sensitivity of available tests; however, these assays could in the future be enhanced by using reactive drug metabolites in place of the parent drug. Patients may experience acute, severe neutropenia, or agranulocytosis, and develop symptoms including fever, chills, sore throat and muscle/joint pain [14]. Diagnosis can be difficult, especially during the COVID-19 pandemic, but timely recognition is critical because, if left untreated, there is an increase in mortality.

Some knowledge regarding the thyroid can be gathered from a previous coronavirus pandemic. The severe acute respiratory syndrome (SARS) epidemic started in November 2002 and spread worldwide. SARS is an infectious condition caused by SARS-CoV, a member of the same family Coronaviridae [2, 15]. Several reviews have attempted to review and summarise the pathogenetic mechanisms and organ involvement in SARS. They were predominantly focused on lung pathology but some aspects of thyroid dysfunction have been addressed. SARS-CoV was found in the lung, trachea/bronchus, stomach, small intestine, distal convoluted renal tubule, sweat glands, parathyroid, pituitary, pancreas, adrenal gland, liver and cerebrum, but was not detected in the oesophagus, spleen, lymph node, bone marrow, heart, aorta, cerebellum, thyroid, testis, ovary, uterus or muscle [16].

In a group of prospectively recruited 61 SARS survivors (with no pre-existing endocrine conditions and investigated at 3/12 after recovery), a reversible hypophysitis or direct hypothalamic effect cased by the virus was suggested. Four (6.7%) SARS patients were reported to be biochemically hypothyroid, including three cases with central hypothyroidism (2/3 were also hypocortisolaemic) and one with primary hypothyroidism: 24 (39.3%) patients had evidence of hypocortisolism and 2 (3.3%) of them also had transient subclinical thyrotoxicosis [17, 18].

Low serum triiodothyronine and thyroxine levels, commonly found in patients with SARS, have also been reported. In 48 SARS patients, serum fT3 and fT4 levels were decreased in the acute phase in 94% and 46%, respectively, whereas during the convalescent phase of the disease in 90% and 38%, respectively. The degree of decrease in fT3 correlated with disease severity. Serum thyroid-stimulating hormone (TSH) concentration in patients with SARS was also significantly decreased in comparison to control group, again suggesting central hypothyroidism [15, 19]. However, autopsies of five SARS cases reviled follicular epithelial damage in the thyroid gland as well, with large numbers of cells exfoliated into the follicle and undergoing apoptosis [15].

The reduced TSH level reported by Wang et al. [15] in patients with SARS cannot be explained by the destruction of follicular epithelium and may relate to the pituitary dysfunction reported by Leow et al. [17], but also it is likely that this implies a manifestation of the ‘sick euthyroid’ syndrome observed in severely ill patients, which is often diagnosed in an intensive care units (ICU) setting. Multiple mechanisms have been suggested to contribute to the development of this condition, including alterations in the iodothyronine deiodinases, TSH secretion, thyroid hormone binding to plasma protein, transport of thyroid hormone in peripheral tissues, and thyroid hormone receptor activity changes. This syndrome may be a form of physiologic adaptation and/or pathologic response to acute illness. The underlying cause for these alterations still remains unclear. Currently available data do *not* provide evidence of a clear benefit of treatment using thyroid hormones in the euthyroid sick syndrome, although on-going clinical trials are focusing on new management strategies to explore whether restoration of normal serum thyroid hormone levels improves patients prognosis and clinical outcomes [20]. At present, there are no publications on euthyroid sick syndrome in COVID-19 patients.

In a recently published series of COVID-19 critically ill COVID-19 patients admitted to the ICU, no pre-existing thyroid problems were reported (even though the majority of patients had chronic illnesses before their admission to intensive care, most commonly it was diabetes mellitus and chronic kidney disease), and it seems that no new thyroid issues have been reported during the COVID-19 illness in this patients cohort [21]. While it is possible that combined hypocortisolism and hypothyroidism might explain the wide range of non-specific symptoms found in recovered patients with SARS (which has been referred to as post-SARS sickness syndrome) [17, 18], abnormal thyroid function in severely compromised patients with COVID-19 should be investigated (and where appropriate, treated) similar to other ITU patients.

In conclusion, there are no data available currently to suggest that patients with AITD remain at higher risk of COVID-19, and it seems likely that most asymptomatic and mildly affected patients should be treated as in normal times. While the risks of anti-thyroid drugs remain, they are probably not directly germane to such patients who should be managed in the usual manner. For many, indeed most patients, some delay in routine blood testing should not cause concern, and patients should be reassured. In patients severely affected by COVID-19, changes in thyroid function may relate to a ‘sick euthyroid’ syndrome, but there may be specific thyroid-related damage which requires further investigation. Further information regarding COVID-19 and thyroid patients has just been published in ETA Public Health Board statement (www.euthyroid.com).
